# Age distribution of patients with advanced non-melanoma skin cancer in the United States

**DOI:** 10.1007/s00403-013-1357-2

**Published:** 2013-04-21

**Authors:** Stacey Dacosta Byfield, Diana Chen, Yeun Mi Yim, Carolina Reyes

**Affiliations:** 1 OptumInsight, 12125 Technology Drive MN002-0258, Eden Prairie, MN 55344 USA; 2 Genentech, Inc., 1 DNA Way, South San Francisco, CA 94080 USA

**Keywords:** Non-melanoma skin cancer, Metastasis, Incidence, Prevalence

## Abstract

The epidemiology of non-melanoma skin cancer (NMSC) is not well understood due to exclusion from most US cancer registries. Patients with at least two claims with a NMSC diagnosis (ICD-9-CM 173.xx) at least 60 days apart, or at least one claim for a NMSC-specific treatment from 1/2010 to 12/2010, were identified from a large US commercial insurance claims database and grouped into one of three cohorts: metastatic (MET), locally advanced (LA), or “all other”. MET patients had at least two claims with a metastasis code at least 30 days apart. LA patients had at least two visits to a medical oncologist, one diagnostic imaging service, two radiation therapy services, or one visit to two or more physician specialties. Remaining patients were “all other”. Incidence and prevalence of NMSC were calculated from among the total number of persons continuously enrolled in the plan during the study period and standardized to the 2010 US population. From among 6,610,256 patients, there were 47,451 incident cases of NMSC (MET *n* = 16, LA *n* = 387, all other *n* = 47,048). The age-adjusted incidence rate of 693 per 100,000 persons (2010 population) approximates to 2,139,535 total NMSC cases in the US (0.7 % of population). 671 prevalent cases had advanced disease (MET *n* = 43, LA *n* = 628); an age-adjusted rate of 0.6 and 10 per 100,000 US persons equivalent to 1,993 and 29,841 MET and LA cases, respectively. Although NMSCs rarely progress, the number of patients with advanced disease is significant. Further studies to determine proportions of advanced NMSC by subtype are needed.

## Introduction

Non-melanoma skin cancer (NMSC) is the most common cancer in the United States and is steadily increasing in annual incidence. A limited number of publications exist on the incidence and prevalence of NMSC due to exclusion from US cancer registries. Rogers et al. [13] estimated that 2,152,500 persons in the United States were treated for 3,507,693 NMSCs in 2006. In the Medicare population, the number of annual incident NMSC cases increased an average of 4.2 % from 1992 to 2006 [13]. Incidence rates are often highest for men and in the older age categories [1–3], suggesting a cohort effect of increasing ultraviolet exposure in successive, younger generations [2, 3]. NMSC also poses significant burden to the US healthcare system. Although mortality is low, the high volume of cases places NMSC among the most costly of cancer malignancies. In an analysis of Medicare billing claims in 1992–1995, NMSC accounted for 4.5 % of the $13 billion spent on cancer management [8].

The major subtypes of NMSC are basal cell carcinoma (BCC) and squamous cell carcinoma (SCC). Studies at the local state level or ex-US registries estimate the proportion of BCC patients to be 70–80 % and SCC patients to be approximately 20 % of NMSC cases [1, 6, 9]. Less than 1 % of NMSC cases are other skin malignancies, including Merkel cell carcinomas and various sarcomas. In a small subset of BCC patients, invasion to subcutaneous structures can lead to locally advanced disease and may result in substantial morbidity due to common BCC site locations on the head and neck. Metastatic BCC is rare, with an estimated incidence between 0.00281 and 0.5 % [11]. SCC has a higher metastatic rate, with published estimates of 0.5–16 % [5]. Of the estimated 700,000 cases of SCC diagnosed annually in the United States, approximately 2,500 result in death [14]. SCCs are at least twice as frequent in men as in women [15], and occur mostly commonly in areas frequently exposed to ultraviolet rays [12], but may occur on any area of the body [15]. Estimating the exact incidence of BCC and SCC will be possible with the histology-specific billing codes which became available in October 2011 [4].

In an effort to better comprehend the epidemiology of NMSC, this study characterizes the incidence and prevalence of locally advanced and metastatic NMSC in a privately insured population.

## Materials and methods

### Data source

This study used administrative claims data from a large national US managed care health plan affiliated with Optum. The proprietary research claims database, the Optum Research Database (ORD), contains data on commercially insured and Medicare Advantage enrollees and includes ~14 million members annually. Member coverage is diverse reflecting the geographic distribution of the nationwide health insurer (12 % West, 23 % Midwest, 37 % South, and 29 % Northeast) and similar to the US census age distribution for gender and age groups <65 years [16]. The ORD includes demographic and enrollment data, pharmacy claims, and medical claims that provide data on services, procedures, and the accompanying diagnoses. Medical claims include diagnosis codes recorded with International Classification of Disease, Ninth Edition, Clinical Modification (ICD-9-CM), procedures recorded with ICD-9 procedure codes, current procedural terminology (CPT) codes, or healthcare common procedure coding system, and revenue codes.

The study data were de-identified and data were accessed in accordance with the Health Insurance Portability and Accountability Act [7] and, therefore, institutional review board approval was not required for this study.

### Patient selection

Commercial and Medicare Advantage health plan members who had a diagnosis of NMSC or “other specified (congenital) anomalies of the skin” captured by the ICD-9-CM code 173.xx were identified from the ORD. To be included in the final study sample, patients had to meet the following inclusion criteria: (1) evidence of NMSC defined as the presence of at least two claims with a diagnosis of NMSC (ICD-9-CM 173.xx) in any position separated by a minimum of 60 days from 01 January 2010 to 31 December 2010; OR at least one claim for a treatment associated with skin cancer management and a diagnosis of NMSC in the primary position on the same claim; (2) continuous enrollment in either a commercial or Medicare Advantage health plan during 2009 and 2010; (3) no evidence of other primary cancers during the study period. Diagnosis codes for other primary cancers included ICD-9-CM codes 140.xx-172.xx, 174.xx-195.xx, 199.xx-209.xx. The presence of at least two claims at least 60 days apart with the same three-digit ICD-9 codes in any position was considered evidence of an additional primary cancer.

From among the patients included in the study sample, three mutually exclusive cohorts were identified: metastatic NMSC patients (MET), locally advanced NMSC patients (LA), and all others (DaCosta-Byfield S, Chen DM, Ramanan DD. Cost and patterns of care among patients with advanced NMSC; ASCO Annual Meeting, June 3–7, 2011, Chicago). MET NMSC patients had at least two claims, at least 30 days apart, with a diagnosis for metastasis (ICD-9-CM = 196.xx–198.xx) in any position during 2010. Since stage of disease is not captured in claims data, an algorithm based on clinical expert opinion attempted to capture procedures and health care utilization associated with NMSC patients who develop advanced disease. LA NMSC patients had at least two claims with diagnosis code 173.xx in any position for (1) medical oncology office visits or outpatient visits on separate days, or (2) diagnostic imaging services, or (3) radiation therapy on separate days, or (4) at least one outpatient or office visit to two or more physician specialties: medical oncology, radiation oncology, or otolaryngology (ENT). A diagnosis for lesion location on lip (ICD-9 173.0), eyelid (ICD-9 173.1), ear (ICD-9 173.2), face (ICD-9 173.3) or scalp, and neck (ICD-9 173.4) was required on the same claim for the radiation oncology visit and the otolaryngology visit. Any 173.x code could have been on the claim for the medical oncology visit. Patients who did not meet criteria for either the MET or the LA population were considered to have early stage disease and were categorized in the “all other” cohort.

### Analysis

To determine incidence estimates during 2010, patients with evidence of NMSC during 2009 were excluded. The prevalence of advanced disease was determined from the frequency of patients with metastatic or LA disease in 2010 regardless of evidence of NMSC in 2009.

Age-adjusted incidence rates of NMSC and prevalence of advanced disease were calculated per 100,000 persons continuously enrolled in the health plan during 2009 and 2010, and standardized to the 2010 US population.

## Results

**Fig. 1:**
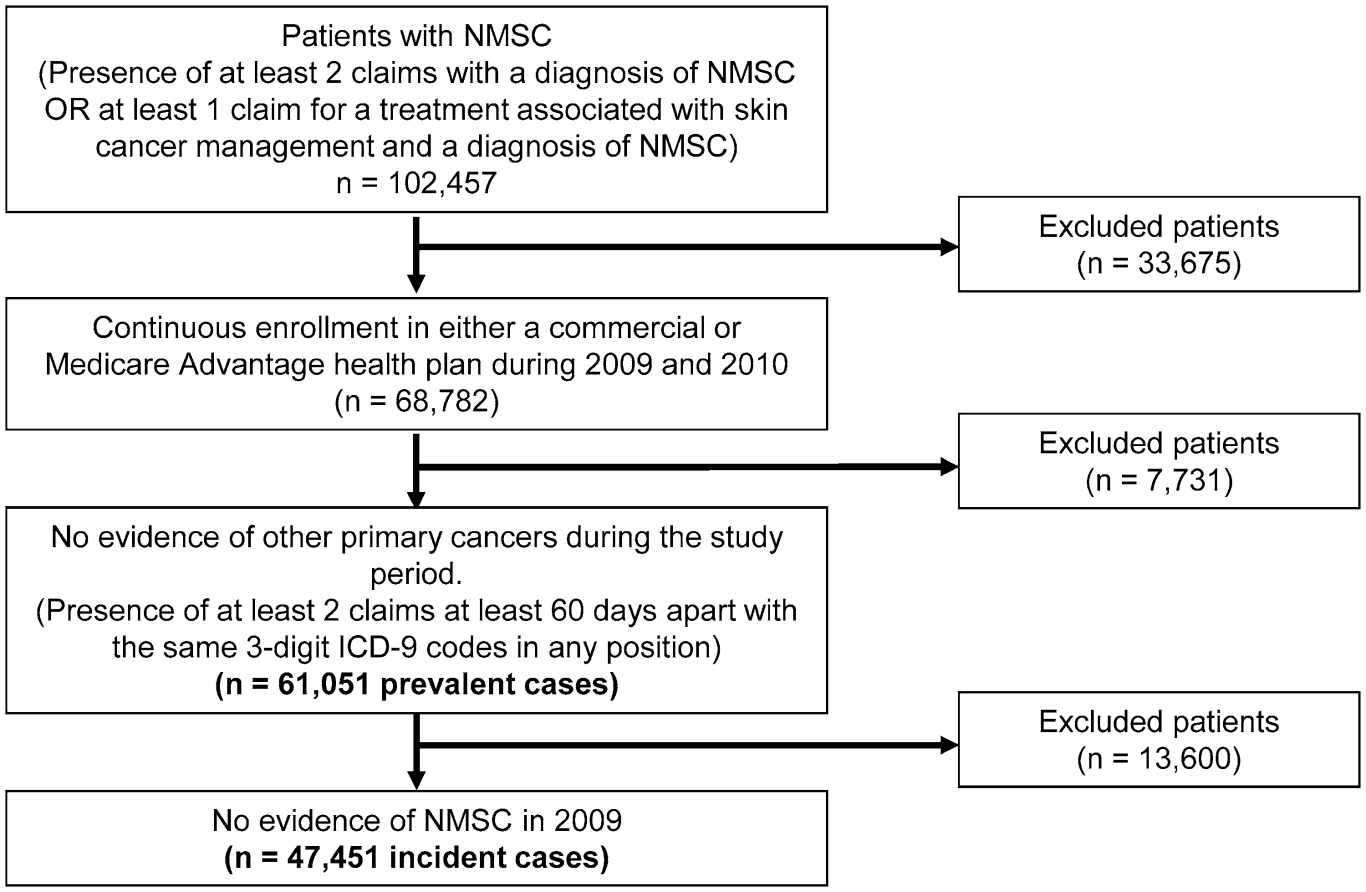
Sample selection and attrition

From among the 6,610,256 health plan enrollees with continuous enrollment from 1 January 2009 to 31 December 2010, a total of 68,782 patients had evidence of NMSC during 2010 (Fig. 1). After excluding 7,731 patients with evidence of other primary cancer, there were a total of 61,051 patients with evidence of NMSC during 2010. Among these, 671 patients had evidence of advanced disease (MET *n* = 43 and LA *n* = 628) during 2010 and were considered the prevalent population. In addition, a total of 47,451 patients had no evidence of NMSC during 2009 and were considered incident cases (MET *n* = 16, LA *n* = 387, all other *n* = 47,048).

Among the incident cases, the median age was 69 years for the MET cohort, 74 for the LA cohort, and 64 for the “all other” cohort. As expected, most NMSC cases were male and older in age, regardless of cohort (Table 1). Among LA and MET patients, 85 and 95 %, respectively, were 55 years or older. In addition, whether incident or prevalent cases; more than 60 % of the LA patients were Medicare advantage enrollees compared to less than half of the MET patients, though the small sample size of metastatic cases makes this difficult to interpret. Overall, the distribution of both incident and prevalent patients in large geographic regions of the Midwest, Northeast, South, and West was similar to the distribution of enrollees in the health plan overall, with the largest percentages of patients residing in the South followed by the Midwest.

**Table 1:** Demographic characteristics of total enrollees and NMSC patients with continuous enrollment in 2009 and 2010

	Total enrollees (*n* = 6,610,256)		Incident cases in 2010								Prevalent cases in 2010—patients with advanced disease			
			Total cases (*n* = 47,451)		Metastatic (*n* = 16)		Locally advanced (*n* = 387)		All other (*n* = 47,048)		Metastatic (*n* = 43)		Locally advanced (*n* = 628)	
	*n*	%	*n*	%	*n*	%	*n*	%	*n*	%	*n*	%	*n*	%
Age categories														
0–17	1,433,250	22	37	<1	0	0	1	<1	36	<1	0	0	1	<1
18–24	445,109	7	57	<1	0	0	1	<1	56	<1	0	0	1	<1
25–34	797,610	12	551	1	0	0	0	0	551	1	0	0	0	0
35–44	1,063,028	16	2,761	6	0	0	14	4	2,747	6	0	0	17	3
45–54	1,174,338	18	7,935	17	1	6	33	9	7,901	17	1	2	49	8
55–64	896,985	14	12,572	26	6	38	61	16	12,505	27	16	37	94	15
65–74	427,895	6	10,673	22	3	19	86	22	10,584	22	8	19	140	22
75+	372,041	6	12,865	27	6	38	191	49	12,668	27	18	42	326	52
Male gender	3,214,386	49	25,888	55	11	69	222	57	25,653	55	36	84	391	62
Insurance														
Commercial	6,001,709	91	31,436	66	10	63	147	38	31,277	66	24	56	235	37
Medicare advantage	608,547	9	16,017	34	6	38	240	62	15,771	34	19	44	393	63
Region														
Northeast	692,892	10	4,428	9	3	19	42	11	4,383	9	5	12	64	10
Midwest	1,661,453	25	10,998	23	5	31	98	25	10,895	23	15	35	156	25
South	3,261,977	49	24,647	52	5	31	204	53	24,438	52	15	35	348	55
West	990,445	15	7,360	16	3	19	43	11	7,314	16	8	19	59	9
Other/unkown	3,489	<1	18	<1	0	0	0	0	18	<1	0	0	1	<1

The age-adjusted incidence of NMSC (Table 2) overall was 693 per 100,000 people standardized to the 2010 US population, which is equivalent to approximately 2,139,536 NMSC cases (0.7 % of the US population). Incidence rates of metastatic and locally advanced disease were 0.238 and 5.926 per 100,000 persons, equivalent to 734 (0.03 % of cases) and 18,297 patients (0.9 % of US cases) in 2010, respectively (Table 2). Incidence rates were highest among individuals aged 75 years or older at a calculated rate of 208 per 100,000 persons.

**Table 2:** Rates of NMSC s during 2010

	Incident cases in 2010						Prevalent cases in 2010—patients with advanced disease			
	Total cases (*n* = 47,451)		Metastatic (*n* = 16)		Locally advanced (*n* = 387)		Metastatic (*n* = 43)		Locally advanced (*n* = 628)	
	Rate^a^	95 % CI	Rate^a^	95 % CI	Rate^a^	95 % CI	Rate^a^	95 % CI	Rate^a^	95 % CI
Age categories										
0–17	0.62	0.44–0.85	–	–	0.02	0.00–0.09	–	–	0.02	0.00–0.09
18–24	1.27	0.96–1.65	–	–	0.02	0.00–0.12	–	–	0.02	0.00–0.12
25–34	9.19	8.42–9.96	–	–	–	–	–	–	–	–
35–44	34.55	33.26–35.84	–	–	0.18	0.10–0.12	–	–	0.21	0.12–0.34
45–54	98.50	96.33–100.67	0.01	0.00–0.07	0.41	0.28–0.58	0.01	0.00–0.07	0.61	0.45–0.80
55–64	165.62	162.72–168.51	0.08	0.03–0.17	0.80	0.61–1.03	0.21	0.12–0.34	1.24	1.00–1.52
65–74	175.42	172.09–178.75	0.05	0.01–0.14	1.41	1.13–1.75	0.13	0.06–0.26	2.30	1.92–2.68
75+	207.81	204.22–211.40	0.10	0.04–0.21	3.09	2.65–3.52	0.29	0.17–0.46	5.27	4.69–5.84
Total per 100,000 persons	692.977		0.238		5.926		0.645		9.665	
Estimate of total persons with NMSC in 2010 in the US	2,139,535		734		18,297		1,993		29,841	

^a^Age-adjusted incidence per 100,000 people (standardized to the 2010 US population)

A total of 671 prevalent NMSC cases with advanced disease were detected in 2010 (MET *n* = 43, LA *n* = 628). The age-adjusted prevalence was 0.645 and 9.665 per 100,000 people standardized to the 2010 US population which is equivalent to 1,993 and 29,841 advanced NMSC cases, respectively, in the United States in 2010.

## Discussion

Although NMSCs, such as BCC and SCC, are the most common skin cancers in the United States, their epidemiology is poorly understood due to the lack of reporting in US cancer registries. Furthermore, NMSC subtypes cannot be accurately tracked because there are no ICD-9-CM codes that uniquely classify BCC malignancies, SCC, or “other malignancies of the skin”.

This retrospective study characterized the age distribution and epidemiology of NMSC patients and found that incidence was approximately 0.7 %, which is equivalent to 2,139,536 NMSC cases in the US in 2010. This incidence rate is consistent with the most recent published estimate of 2,152,500 patients treated for NMSC during 2006 [13]. As in previous reports, incidence rates were highest in the older age categories [1–3], and men were more often diagnosed with NMSC. Among the incident NMSC cases, 0.03 and 0.9 % were metastatic and locally advanced, respectively. Previously reported estimates of metastatic disease have wide ranges from 0.0028 to 0.5 % for BCC [11], and from 0.5 to 16 % for SCC [5]. The estimate for the aggregate NMSC population in this study, is therefore within range for BCC and low for SCC; however, this is difficult to interpret since the proportion of BCC and SCC in locally advanced and metastatic cases is not known. In addition, SCC has a slightly higher metastatic potential than BCC and likely constitutes greater than the estimated 20 % of NMSC once the disease progresses. Studies investigating more precise estimates by unique skin cancer diagnosis are warranted.

It must be noted that there are certain study limitations associated with this study and with claims data, which are collected for the purpose of payment and not research. In this study, the indicated ICD-9 code (ICD-9-CM 173.xx) is not specific for any of the NMSC skin cancers, but instead captures all NMSCs. However, BCC is known to be the most common malignancy found in this category [6], accounting for approximately 80 % of all newly diagnosed NMSC cases. Second, the presence of a diagnosis code on a medical claim is not positive presence of disease, as the diagnosis code may be incorrect or may not precisely capture the diagnosis of interest or the extent of disease. Metastatic disease is likely under-reported in claims data given it is not a requirement to precisely code primary versus secondary malignancies. Further, since stage of disease is not captured in claims data, an algorithm based on expert clinical opinion and treatment guidelines, but not yet validated, was created to characterize locally advanced versus metastatic and early stage (“all other”) disease. In addition, the exclusion of prevalent cancer patients with evidence of other cancers during the study period (approximately 11 % of patients as noted in the “Methods” Section), may underestimate the cases of NMSC. Finally, the proportions of cases identified in the claims data from a single health insurer may not be generalizable to the entire US population. However, the estimate of incident NMSC presented here is consistent with previously published estimates [10, 13].

In summary, this study is among the few analyses that attempt to characterize the epidemiology of locally advanced and metastatic rates of NMSC. While the patient population with advanced disease is relatively small, an unmet medical need exists due to high morbidity and limited therapeutic options. NMSC truly poses a public health burden, as the annual number of incident NMSC cases exceeds the combined total of annual incident cases of colon, prostate, lung, breast, and prostate cancer. As NMSC incidence rates continue to rise, it is likely that occurrence of advanced disease will also increase. The recent availability of BCC- and SCC-specific ICD-9 codes in October 2011 warrants further research as it will enable better characterization of the epidemiology of NMSC and the histologic subtypes of BCC and SCC.
